# Recent Advances in Adsorption–Photocatalytic Removal of Pharmaceuticals From Water Using Hybrid Ion‐Adsorbent Photocatalyst

**DOI:** 10.1002/wer.70368

**Published:** 2026-03-30

**Authors:** Pauline Ncube, Azwifunimunwe Tshikovhi, Sisonke Sigonya, Olayemi Fakayode, Bakang Moses Mothudi, Mokgaotsa Jonas Mochane

**Affiliations:** ^1^ Department of Chemistry University of South Africa Pretoria South Africa; ^2^ Centre for Materials Science University of South Africa Pretoria South Africa; ^3^ Department of Physics University of South Africa Pretoria South Africa

**Keywords:** adsorption, emerging pollutants, environment, pharmaceuticals, photocatalysis, wastewater remediation

## Abstract

The remediation of pharmaceutical drugs (PHACs) in the aqueous environment has been one of the major challenges facing the global community for decades. The risk associated with these compounds is their ubiquitous persistence in the environment, posing potential human health risks and ecotoxicological effects. The combined adsorption–photocatalysis process has proven successful in eliminating a wide range of emerging pollutants (EPs), including PHACs. The current review provides updated literature on the recent advancements in adsorption–photocatalytic removal of PHACs from wastewater using hybrid ion‐adsorbent photocatalytic systems. Process performance has been discussed. Limitations have been identified, and possible solutions are proposed to overcome or reduce them. The exemplary investigations of this review would clearly equip the research community with the updated data associated with research gaps for further research aimed at developing efficient ion‐adsorptive photocatalysts, overall process improvements, and potential scale‐up operations. This review is crucial, given that adsorption–photocatalysis has shown promising results in eliminating EPs, including PHACs in wastewater, yet practical applications remain elusive.

## Summary

- Ion‐adsorption photocatalysis improves pharmaceutical removal from water.
- Adsorption enhances pollutant uptake and photocatalytic degradation efficiency.
- Hybrid photocatalyst design improves catalyst selectivity and recovery.
- Stability, fouling, and scale‐up remain key challenges for practical use.

## Graphical abstract

This review focuses on recent advancements in hybrid ion adsorbent–photocatalysts for the removal of pharmaceuticals from water, highlighting improved performance, catalyst innovations, mechanistic insights, scalability barriers, and future perspectives.

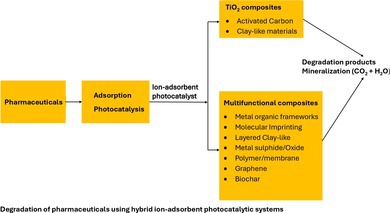

**Abbreviations**
ACM: acetaminophen
AMO: amoxicillin
CBZ: carbamazepine
CEF: ceftriaxone
CIP: ciprofloxacin
CTC: chlortetracycline
DCF: diclofenac
DOX: doxorubicin
DXC: doxycycline
E2: 17b‐estradiol
FBP: flurbiprofen
IBU: ibuprofen
LVF: levofloxacin
MB: methylene blue
NPX: naproxin
NOR: norfloxacin
OFL: ofloxacin
OXC: oxytetracycline
PHACs: pharmaceutical drugs
PRL: propranolol
PST: pesticides
SMX: sulfamethoxazole
TC: tetracycline
TCN: triclosan

## 1 Introduction

Pharmaceutical compounds have risen to become new classes of water contaminants due to their potential detrimental effects on human health and the environment at large. Unfortunately, the use of these compounds cannot be limited due to their beneficial human and veterinary therapeutic applications. Moreover, the continuous emergence of new diseases and growing populations demand the development of new PHACs and increased production of the existing ones. That makes their massive release into the aquatic environment through excretory routes and wastewater discharges from production facilities imminent. Several authors have reported increasing concentrations of PHACs in various aqueous matrices across the globe (Adhikari et al. 2023; Netshithothole et al. 2024; Khasawneh and Palaniandy 2021). Some PHACs have been classified as emerging pollutants (EPs) due to their refractory nature and resistance to conventional wastewater treatment, highlighting the urgent need for effective and sustainable alternative treatment methods.

Adsorption and photocatalysis have gained a lot of attention and recognition for removing water pollutants due to several advantages over traditional treatment methods. Adsorption is particularly attractive for its low cost, simplicity, and the availability of diverse adsorbent materials, including biomass, agricultural waste, clay minerals, and industrial byproducts. However, unmodified adsorbents often exhibit low efficiency, which can be enhanced through physical or chemical modifications (Thue et al. 2018; Li, Huang, et al. 2021). Recent advancements in nanotechnology enabled the development of nano‐adsorbents with superior properties such as high selectivity, surface area, porosity, strong pollutant affinity and in some cases low cost (Hussain, Riaz, et al. 2025). Among these, ion adsorbents such as metal organic frameworks (MOFs), and polymer‐based nanocomposites have raised considerable interest in wastewater remediation, particularly in heavy metal (Hussain, Alam, et al. 2025), and dye removals (Park et al. 2025). Interestingly, recent literature shows growing application of ion‐ adsorbents in PHACs removals (Demiti et al. 2025; Qamer Abbas et al. 2025). These adsorbents use a combination of superior porosity, surface charge and functional groups interactions, to remove PHACs from wastewater (Qamer Abbas et al. 2025). Furthermore, their architecture is customized to match the structure, charge and reactivity of PHACs, facilitating effective pollutant decomposition. However, despite these advancements and advantages, single adsorption processes lack the capability to mineralize PHACs and may result in pollutant phase transfer to sludge waste (Chen et al. 2022). Moreover, safe disposal of contaminated adsorbents can increase treatment costs.

In contrast, photocatalysis presents the potential for complete decomposition of pollutants using light sources such as, solar, infrared or UV light to activate semiconductor catalysts. The technique generates reactive species (e.g., •OH, •O_2_^−^) that oxidize and break down pollutants. High degradation efficiencies, including mineralization, have been reported in other studies (Heidari et al. 2020; Czech et al. 2020; Jabeen et al. 2024) Nonetheless, photocatalysis suffers from limitations such as, low quantum efficiency, rapid charge recombination, and high energy demands, particularly in UV light systems (Jabeen et al. 2024), making it less economically appealing. Current research focuses on developing visible‐light‐active photocatalysts to reduce energy costs and improve solar utilization. However, variations in sunlight intensity across regions and seasons may hinder large‐scale solar‐driven applications.

Combining adsorption with advanced oxidation processes (AOPs), particularly in hybrid adsorption–photocatalysis systems, has demonstrated improved effectiveness for removing EPs, including PHACs. For example, complete removal of amoxicillin (AMO) and ciprofloxacin (CIP) has been achieved using hybrid ion‐adsorptive photocatalysts, TiO_2_/chitosan (TiO_2_‐CS) (Bergamonti et al. 2019) and TiO_2_–hydroxyapatite (TiO_2_‐HA) (Cheikh et al. 2023; Bouyarmane et al. 2021) composites. These findings can be explained by synergistic interfacial interactions between the adsorptive surface and the photocatalyst, which promote rapid pollutant adsorption, suppress electron–hole (e^−^/h^+^) recombination, and enhance charge separation, thereby increasing photodegradation efficiency. In ion‐adsorptive photocatalysts, selective pre‐concentration of charged or polar PHACs via tailored cavities, functional groups, and electrostatic interactions further enhances degradation compared with single‐process systems (Qamer Abbas et al. 2025). This integrated adsorption–photocatalysis overcomes key limitations such as slow kinetics, catalyst deactivation, and incomplete mineralization, while performance can be further improved through surface functionalization, heterojunction engineering, and incorporation of high‐surface‐area carbonaceous materials.

Although adsorption and photocatalysis for water treatment have been discussed in several reviews, they are mostly treated as separate processes. In contrast, this review focuses on ion‐adsorption–photocatalysis systems, emphasizing the synergistic rather than independent features of adsorption and photocatalytic degradation. The review further highlights the mechanistic role of ion adsorption in pre‐concentrating PHACs, promoting interfacial charge transfer, and improving overall photodegradation efficiency. Rather than simply categorizing material types, this work covers a wide range of hybrid systems, organizing them according to their ion‐adsorptive and photocatalytic characteristics. Importantly, the review looks further than performance comparisons by identifying major challenges, such as scalability, material stability, regeneration, and selectivity in complex wastewater matrices, and discussing practical strategies to address them. Overall, the review seeks to establish a clear link between laboratory‐scale developments and their potential for practical wastewater treatment solutions.

## 2 TiO_2_‐Based Adsorption–Photocatalysis

Several metal oxides have been employed, including TiO_2_, Fe_2_O_3_, ZnO, and WO_3_, with TiO_2_ being the most widely used in wastewater remediation (Velempini et al. 2021). Compared with the other metal oxides, TiO_2_ exhibits superior qualities such as high redox power, excellent chemical and photostability, non‐hazardous nature, biocompatibility, capacity to decompose a broad range of pollutants, low cost, and wide availability (Peiris et al. 2021). Nonetheless, the main setback of the TiO_2_ photocatalyst is its broad bandgap, which is reportedly 3.02 eV for the rutile phase and 3.2 eV for the anatase phase, respectively (Abdullah et al. 2023). Higher photoactivation energy, usually in the UV spectrum, is needed for such large bandgaps, thus restricting visible light absorption to a small portion of the solar spectrum. Strategies such as noble metal capping, semiconductor coupling, dye sensitization, and metal/nonmetal doping have been explored to mitigate these challenges (Bergamonti et al. 2019; Bi et al. 2021). Importantly, significant progress has been made in developing visible‐light‐active TiO_2_‐based materials. In some studies, TiO_2_ has been combined with adsorbent materials to achieve simultaneous adsorption and photocatalytic decomposition of organic contaminants in water. TiO_2_‐based ion‐adsorbent photocatalysts for the degradation of PHACs in water remediation are summarized in Table 1.

**TABLE 1:** TiO_2_‐based ion‐adsorptive photocatalytic systems for the degradation of PHACs in water.

Catalyst	Pollutant	Light source	Conditions	Removal (%)	References
Polymer/molecular imprinting					
TiO_2_/CS	AMO	UV/visible	Conc: TiO_2_ 1:100 Time: 2 h	100	(Bergamonti et al. 2019)
MICN‐TiO_2_	CIP SMX	UV	Conc: 40 mg/L Catalyst: 0.2 g/L Time: 6 h		(Li et al. 2020)
MIP‐TiO_2_	DCF	UV	Conc: 1 mg/L Catalyst: 0.4 g/L Time: 10 min	89	(Bi et al. 2021)
TiO_2_/ONLH	PHACs (PRL)	Solar light	Conc: 10 mg/L Catalyst: 0.4 g/L	99	(Zhang, Du, et al. 2021)
Magnetic CNT‐based					
MCNT‐TiO_2_	CBZ SMX	Solar light (simulated)	Conc: 0.15 mg/L Catalyst: 0.1 g/L Time: 30 min		(Awfa et al. 2019)
TiO_2_@ZnFe_2_O_4_/Pd	DCF	Solar light	Conc: 10 mg/L Catalyst: 0.03 g/L Time: 2 h	86	(Ahmadpour et al. 2020)
Activated carbon‐based					
AC‐TiO_2_	CEF	Visible	Conc: 100 mg/L Catalyst: 1 g/L Time: 4 h	100 99 (TOC)	(Abdullah et al. 2023)
AC‐TiO_2_	DCF CBZ SMX	Solar light	Conc: 50 mg/L Catalyst: 0.1 g/L Time: 6 h	100 70 50	(Daou et al. 2020)
AC‐TiO_2_	IBU	UV	Conc: 5‐25 mg/L Catalyst: AC90T10 Time: 4 h	92	(Gu et al. 2019)
APOM/TiO_2_	DCF	UV	Conc: 4 mg/L Catalyst: 0.67 mg/L Time: 120 min	95 84 (TOC)	(Gupta et al. 2021)
Clay‐like materials					
TiO_2_‐HA	CIP	UV	Conc: 1 mg/L Catalyst: 1 g/L Time: 90 min	100 98.5 (TOC)	(Cheikh et al. 2023)
TiO_2_‐HA	OFL CIP	UV	Conc: 20 mg/L Catalyst: 2 g/L Time: 15 min Time: 120 min	100 100	(Bouyarmane et al. 2021)
C‐TiO_2_/zeolite	PHACs PST	Solar light (simulated)	Conc: 0.1 mg/L Catalyst: 0.7 g/L Time: 2 h	≥ 95%	(An et al. 2016)

Abbreviations: APOM: hybrid adsorptive‐photocatalytic oscillatory membrane; CN: g‐C_3_N_4_; CS: chitosan scaffolds; HA: hydroxyapatite; MCNT: magnetic carbon nanotube; MICN: molecularly imprinted carbon nanosheets; MIP: molecularly imprinted polymer; ONLH: oxygen or nitrogen linked heptazine‐base polymer.

Table 1 illustrates the expanding application of TiO_2_‐based adsorption–photocatalytic systems in water remediation to remove pharmaceutical residues, with growing emphasis on ion‐adsorbent–photocatalysts to improve selectivity and efficiency. Notably, polymeric photocatalysts have risen in popularity due to their low cost, customizable surface chemistry, and capability to add charged functional groups that facilitate electrostatic interactions with ionizable PHAC molecules. In one study, 10 common PHACs were successfully destroyed by the TiO_2_/ONLH polymer under natural sunlight, with (propranolol) PRL showing the highest removal (Table 1). Furthermore, the composite displayed stability and reusability potential after multiple treatment (Zhang, Du, et al. 2021). This performance could be explained by the polymer's enhanced visible‐light activity and its capacity to utilize surface interactions to concentrate charged PHACs close to TiO_2_ reactive sites. The practical potential of TiO_2_/ONLH was highlighted by its high performance, good visible light response and toxicity removal in tap water, surface water, and wastewater effluent (Zhang, Du, et al. 2021). However, to validate this potential, future research should include prolonged reusability testing, matrix‐specific interference studies, and life‐cycle assessments.

Recent advancements further highlight ion‐adsorbent photocatalysis through the integration of MIPs with polymeric photocatalysts to improve contaminant selectivity via electrostatic interactions, surface complexation, and tailored recognition binding sites (Singh et al. 2020). Due to their high chemical stability, resistance to extreme conditions, and extended lifespan, MIPs can also be integrated with TiO_2_‐based photocatalysts to produce efficient MIP adsorption–photocatalytic systems (Singh et al. 2020; Yi et al. 2023). Bergamonti et al. (Velempini et al. 2021) obtained complete AMO removal using 3D‐printed chitosan–TiO_2_ (TiO_2_/CS) scaffolds (Table 1). The outstanding performance was attributed to chitosan's functional groups (‐OH, ‐NH_2_), which promoted ion adsorption of PHACs, while the porous 3D structure facilitated mass transfer, surface accessibility, and light utilization (Amaly et al. 2022; Li et al. 2019). Additionally, the scaffolds presented potential as reusable filters and industrial photocatalyst supports (Bergamonti et al. 2019).

In a similar study, MICN‐TiO_2_ composites demonstrated high selectivity and photocatalytic efficiency for CIP removal, even in the presence of competing pollutants (SMX) (Table 1). These results could be ascribed to the imprinted nanolayer, high carbon conductivity, and improved charge separation, which synergistically supported ion‐adsorption photocatalysis (Li et al. 2020). Comparable findings were reported by Bi et al. (2021) for DCF using MIP‐TiO_2_ (Table 1), with the improved removals attributed to enhanced stability and superior photodegradation performance compared with non‐imprinted composites.

**FIGURE 1:**
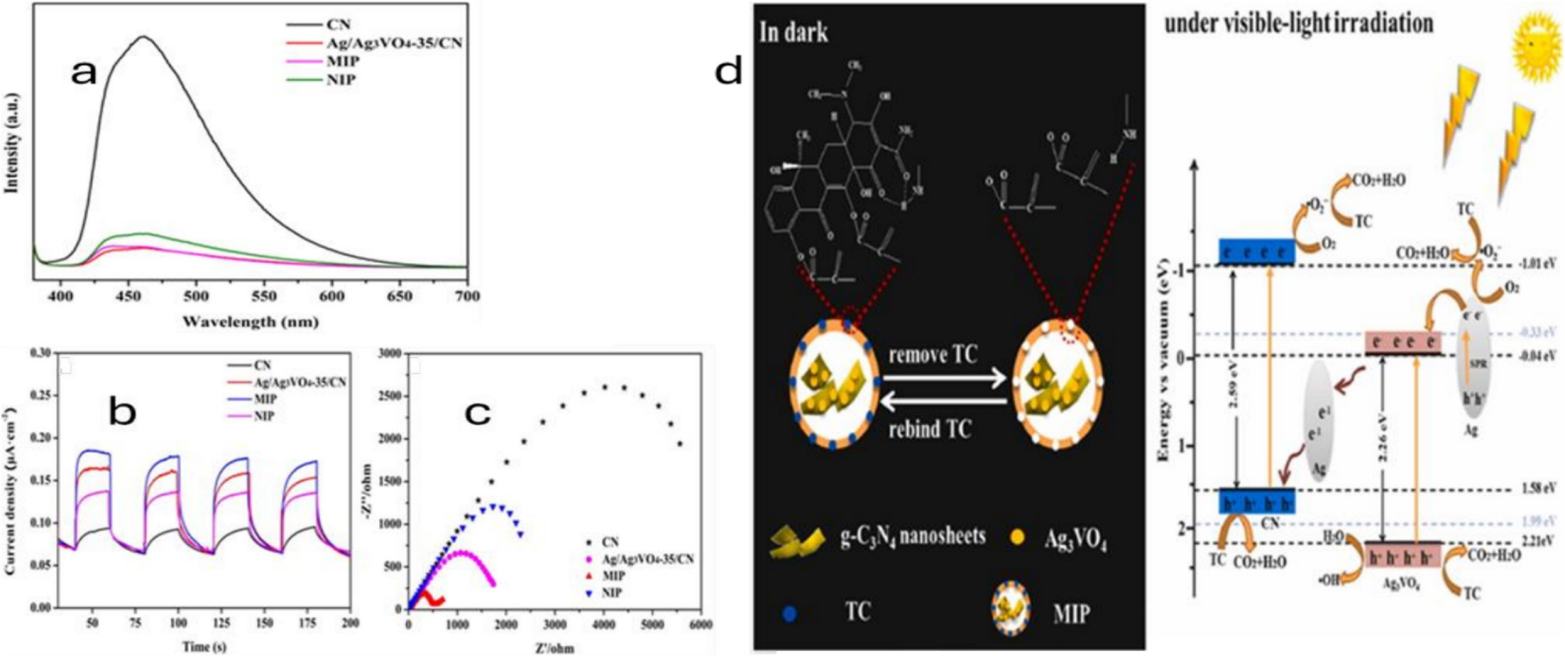
(a) The photoluminescence (PL) spectra of CN, Ag/Ag_3_VO_4_‐35/CN, MIP, and NIP photocatalysts. (b) Transient photocurrent response curves and (c) electrochemical impedance (EIS) spectra of CN, Ag/Ag_3_VO_4_‐35/CN, MIP, and NIP. (d) Proposed mechanism of adsorption–photocatalytic degradation of TC in Z scheme Ag/Ag_3_VO_4_/CN@MIP (Sun et al. 2019). “Copyright permission obtained from ELSEVIER”.

Overall, these findings offer an effective approach for utilizing MIP to simultaneously increase selectivity and photocatalytic activity. However, MIP‐based ion‐adsorbent photocatalysts still face several challenges, including reduced selectivity, particularly when non‐imprinted polymers (NIPs) exhibit high affinity for the template molecule, leading to non‐specific binding. Optimized template‐monomer selection, regulated cross‐linking, and surface‐imprinting techniques that prioritize accessible recognition sites could be useful in mitigating this problem. Limited data on catalyst regeneration and reusability hampers effective evaluations of sustainable applications and scalability (Sun et al. 2019). This limitation is further multiplied by the complex and time‐consuming synthesis of some MIP composites. Future research should focus on standardized reusability tests, stability evaluations, and streamlined production techniques to transform MIP photocatalysts into practical water treatment solutions. The exemplary mechanism of adsorption–photocatalysis using MIP‐based composites is demonstrated in Figure 1.

As illustrated in Figure 1a, the lower PL intensity of Ag/Ag_3_VO_4_‐35/CN@MIP (MIP) compared with CN demonstrates effective suppression of charge recombination. This is affirmed by the higher photocurrent response (Figure 1b) and smaller EIS arc radius (Figure 1c), implying efficient charge generation, separation, and transport. Moreover, MIP exhibits a larger surface area with a pore size distribution of 2–5 nm, suggesting that surface polymerization forms an ordered porous structure that provides additional adsorption sites. These findings can explain the superior adsorption–photocatalytic performance of MIP composites, as demonstrated by the ~90% TC removal reported by Sun et al. (2019).

The proposed degradation pathway (Figure 1d) shows that TC is selectively adsorbed by the MIP through its imprinted cavities during the dark phase. Upon visible light exposure, Ag_3_VO_4_ and CN produce e^−^/h^+^ pairs. A Z‐scheme mechanism is supported when h^+^ transferred from Ag_3_VO_4_ cannot generate •OH (H_2_O/•OH = 2.27 eV) because of the low VB potential of CN (1.58 eV). Under the Z‐scheme, Ag nanoparticles boost charge separation by acting as electron mediators and transporting electrons from the CB of Ag_3_VO_4_ to the VB of CN. While the photoelectrons in CN reduce O_2_ to •O_2_^−^, the remaining h^+^ in Ag_3_VO_4_ oxidize H_2_O to produce •OH. Furthermore, Ag produces extra electrons for O_2_ reduction through surface plasmon resonance. TC degradation is predominantly driven by h^+^ and •O_2_^−^, with •OH playing a supporting role (Sun et al. 2019).

Due to their ease of separation and lower risk of secondary pollution, magnetic nanocomposites have been explored as catalysts for the removal of organic contaminants from water. In these systems, magnetic separation is provided by ferrite components (e.g., Fe_3_O_4_, ZnFe_2_O_4_) incorporated into the CNT framework. The CNTs primarily act as conductive scaffolds that promote electron transfer and adsorption, while the ferrite phase enables rapid post‐treatment recovery of the catalyst under an external magnetic field without the need for filtration or centrifugation (Awfa et al. 2019; Ahmadpour et al. 2020). In a previous study, 86% DCF was removed from water using magnetic TiO_2_@ZnFe_2_O_4_/Pd (Table 1). A similar study employed MCNT‐TiO_2_, under solar light (Table 1) demonstrating faster kinetics and lower energy consumption than single TiO_2_ in the elimination of CBZ and SMX (Awfa et al. 2019). CNTs enhanced ion‐adsorbent photocatalytic activity by acting as electron sinks, facilitating charge separation, increasing surface area, and promoting electrostatic interactions and surface complexation of polar or charged PHACs (Awfa et al. 2019).

**FIGURE 2:**
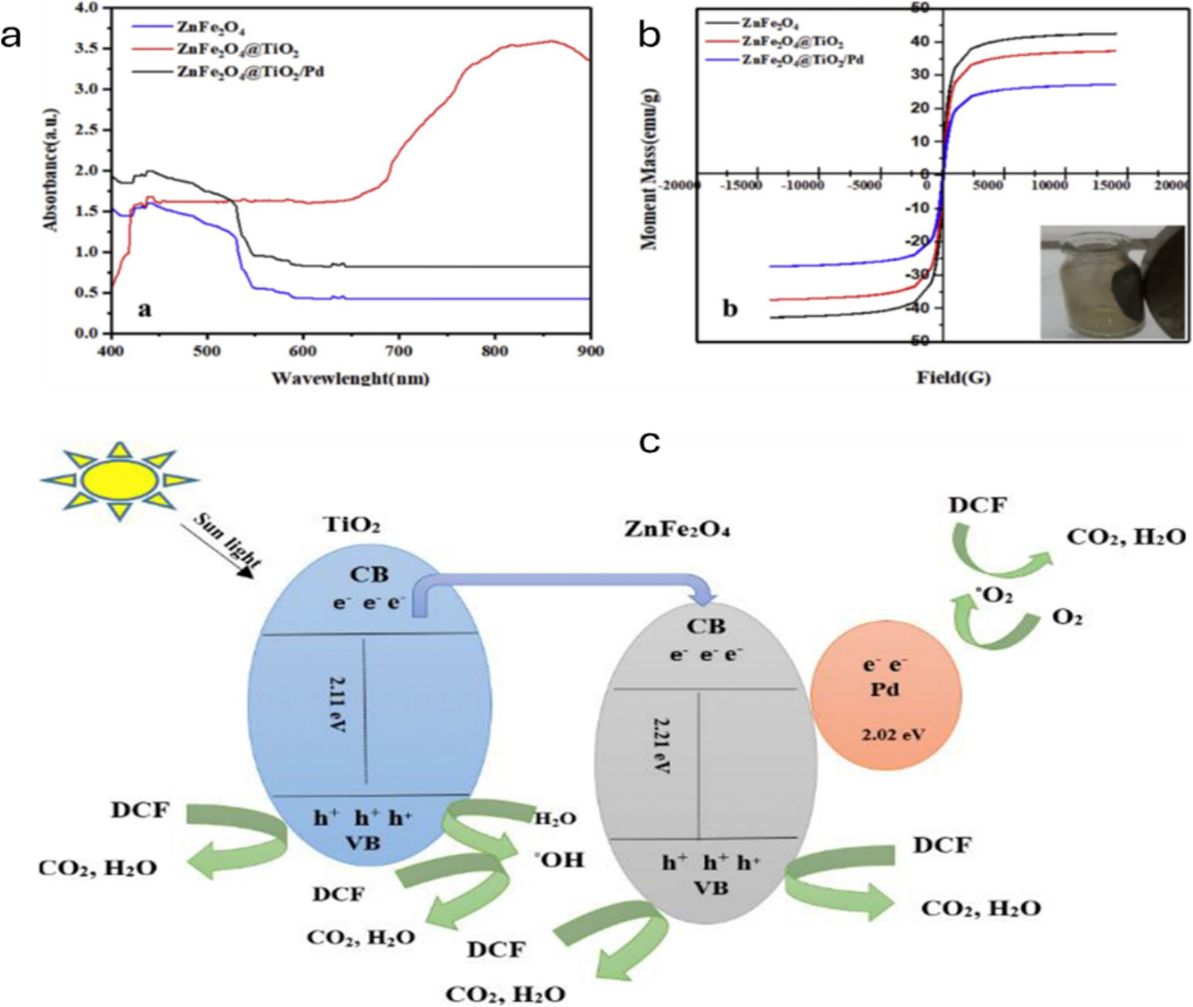
(a) UV–vis DRS spectra. (b) Vibrating sample magnetometry (VSM) of ZnFe_2_O_4_, TiO_2_@ZnFe_2_O_4_ and TiO_2_@ZnFe_2_O_4_/Pd magnetic composites. (c) Proposed mechanism of DCF removal using ZnFe_2_O_4_@TiO_2_/Pd (Ahmadpour et al. 2020). “Copyright permission obtained from ELSEVIER”.

Despite these benefits, magnetic ion‐adsorbent photocatalysts suffer various limitations, such as light shielding by magnetic cores, potential metal leaching, and adsorption competition from natural organic matter (NOM), which might obstruct ion‐binding sites in natural wastewater. Furthermore, the usage of noble metals like Pd restricts scalability and raises material costs. To improve ion adsorption while reducing fouling, future studies should prioritize integrating earth‐abundant cocatalysts, functionalizing catalyst surfaces with zwitterionic or charged groups, and optimizing core–shell structures that balance light penetration and magnetic separation. Such strategies would advance magnetic ion‐adsorbent photocatalysis toward practical water remediation by improving durability, selectivity, and economic viability. The suggested degradation pathway of PHACs using magnetic composites is demonstrated in Figure 2 using the ZnFe_2_O_4_@TiO_2_/Pd system.

The UV–vis (DRS) spectrum (Figure 2a) shows that the ZnFe_2_O_4_, TiO_2_@ZnFe_2_O_4_, and TiO_2_@ZnFe_2_O_4_/Pd composites can be activated by visible light, possibly due to the narrower band gap of the composites. Notably, visible light absorption increased in the presence of Pd (Figure 2a) due to the surface plasmon resonance effects of Pd under visible light irradiation. Pd acted as a mediator facilitating charge migration and plasmon resonance energy transfer between TiO_2_ and ZnFe_2_O_4_ nanoparticles, which promoted charge separation (Ahmadpour et al. 2020). This performance could further account for the observed high DCF degradation (Table 1). Additionally, the N_2_ adsorption–desorption isotherms of TiO_2_@ZnFe_2_O_4_/Pd fitted the mesoporous structure. Mesopores improve light penetration, pollutant diffusion, and active sites accessibility, which promotes adsorption–photocatalysis synergy. Noteworthy, although TiO_2_@ZnFe_2_O_4_/Pd exhibited lower magnetism compared with ZnFe_2_O_4_ (Figure 2b) the composite still lay within the paramagnetic field, allowing easy separation from the solution using an external magnetic field.

Figure 2c shows the proposed mechanism of DCF degradation using magnetic ZnFe_2_O_4_@TiO_2_/Pd. During the dark phase, electrons migrate from Pd to TiO_2_, driven by Pd's higher Fermi level, which brings the photocatalytic system into balance. Upon exposure to solar illumination, TiO_2_ generates e^−^/h^+^ pairs, followed by the formation of •OH and •O_2_^−^ radicals. Pd nanoparticles, with their strong electron‐accumulating ability, accept photoelectrons from TiO_2_, modulating the composite's reduction potential. The cumulative effect of this electron transfer is reduced charge recombination and extended charge carrier lifespan, resulting in enhanced DCF degradation (Ahmadpour et al. 2020).

In a related study on the removal of a mixture of PHACs (CBZ, DCF, SMX) using AC‐TiO_2_, DCF was completely removed (Table 1) and exhibited the highest adsorption capacity (Q_max_ = 154 mg g^−1^) (Daou et al. 2020). These results were ascribed to the large pore volume and surface area of the bio‐derived AC (nut shells), which facilitated selective ion adsorption and close contact with TiO_2_ nanoparticles (Daou et al. 2020). This promoted efficient interfacial charge transfer and minimized electron–hole recombination, resulting in enhanced photocatalytic performance. Furthermore, the use of agricultural waste as a precursor for AC highlights the cost‐effectiveness and sustainability of ion‐adsorbent photocatalysts, which aligns with circular economy principles.

A hybrid system integrating AC and TiO_2_ within an adsorptive‐photocatalytic oscillatory membrane (APOM/TiO_2_) achieved 95% DCF degradation and 84% TOC removal under UV light (Table 1). This performance could be explained by the combined effects of ion‐adsorption, photocatalysis, and membrane‐assisted flow modulation. However, prolonged radiation exposure (~100 h) resulted in reduced performance, possibly due to AC surface saturation and the accumulation of transformation products (TPs), which suppressed TiO_2_ activity (Gupta et al. 2021).

**FIGURE 3:**
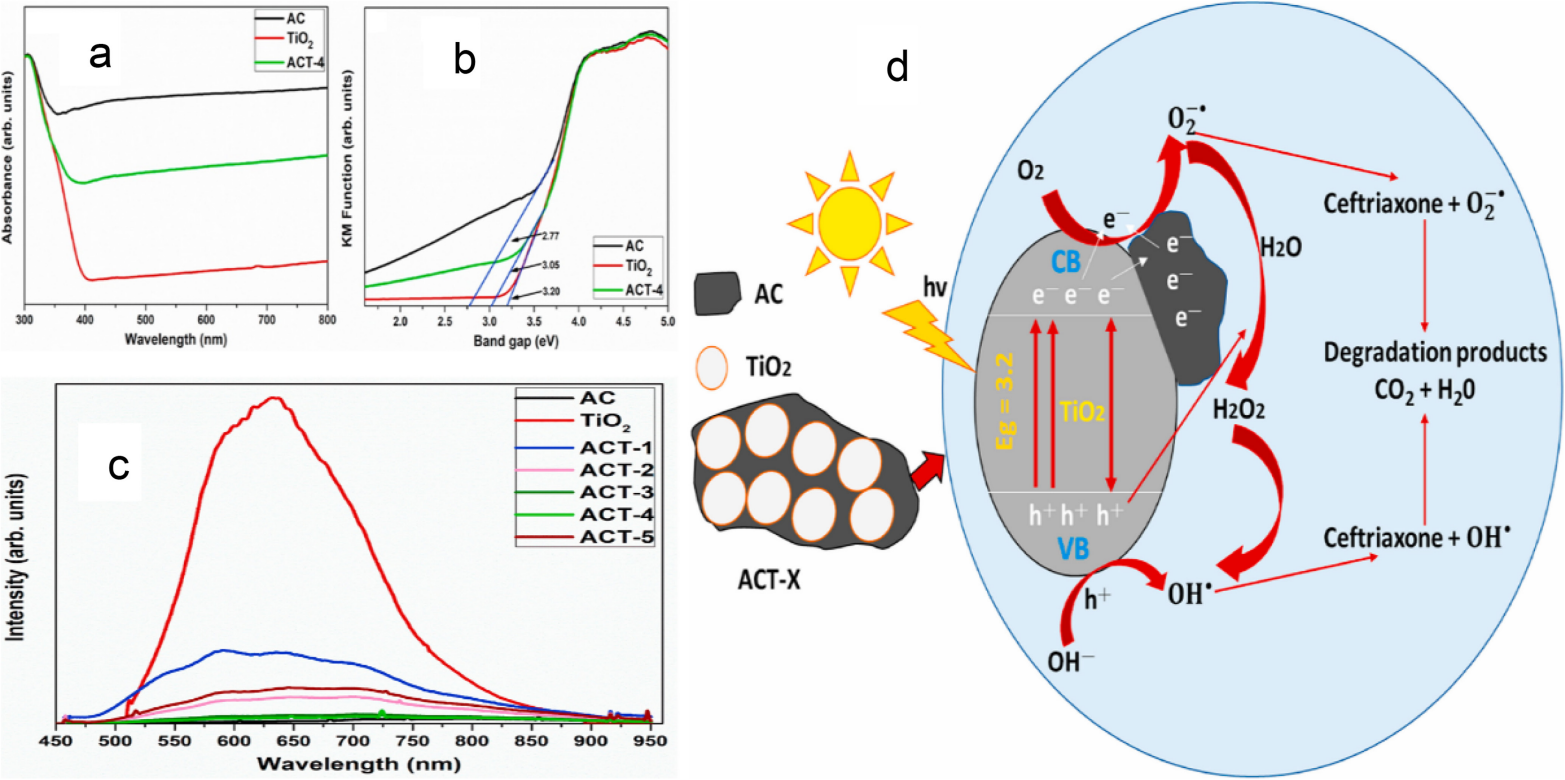
(a) UV–vis DRS spectra. (b) Energy band gap. (c) PL emission spectra of AC, TiO_2_, and ACT‐X samples. (d) Adsorption–photocatalytic degradation mechanism of CEF using AC‐TiO_2_ composite (Abdullah et al. 2023). “Copyright permission obtained from ELSEVIER”.

Expanding on the AC‐TiO_2_ approach, Abdullah et al. (2023) reported complete degradation of CEF and 99% total organic carbon (TOC) removal, while 92% IBU was eliminated in a similar study (Table 1). Additionally, over 80% removal efficiency was maintained after multiple reuse cycles, illustrating the composites' stability and reusability. This high catalytic efficiency could be attributed to the ion‐adsorptive concentration of charged PHACs on AC functional groups, which boosts their availability at TiO_2_ active sites. The synergistic effects of a lower band gap (3.05 eV) expanded visible light absorption, and inhibited charge recombination, further supporting effective ion‐adsorption photocatalysis (Abdullah et al. 2023). Figure 3 demonstrates PHACs removal using an AC‐based adsorptive‐photocatalytic system.

Figure 3a shows that the presence of AC in the AC‐TiO_2_ composite (ACT‐4) enhanced light absorption in the visible spectrum. This is further confirmed by the lower band gap (3.05 eV) of the ACT‐4 compared with pristine TiO_2_ (Figure 3b). During synthesis, the AC and TiO_2_ in the ACT‐4 composite may have interacted to form Ti–O–C linkages, resulting in the development of new heterojunction frameworks or defects that improved light absorption, charge carrier separation and transfer to the AC surface (Cheikh et al. 2023). It is important to mention that AC also provides an adsorptive surface for pollutant adsorption, which boosts pollutant removals by adsorption–photocatalysis. The degradation of CEF using AC‐TiO_2_ (Figure 3) proceeds according to Equations (1)–(6) (Abdullah et al. 2023).

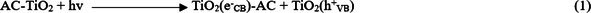

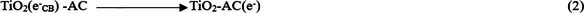

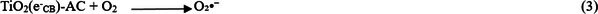

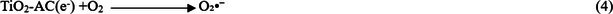

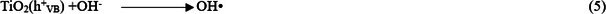

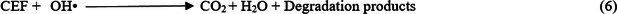

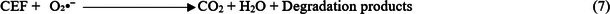

The synergistic relationship between adsorption and photocatalytic degradation in CEF elimination is shown by Equations (1)–(7). By concentrating CEF molecules at the catalyst surface during initial adsorption, photogenerated reactive species can interact with the catalyst more effectively (Equations 1–3). Simultaneous photodegradation of the adsorbed or surface‐close CEF molecules continuously replenishes active adsorption sites, thus preventing surface saturation and maintaining high removal efficiency (Equations 4–7). Additionally, during degradation, AC can function as a conductive support, enhancing the interaction between TiO_2_ and CEF substrates (Abdullah et al. 2023). Overall, the total elimination of CEF surpasses that of individual photocatalysis or adsorption, highlighting synergistic effects.

Collectively, these studies demonstrate the promising potential of AC‐TiO_2_ composites in water remediation, presenting enhanced efficiency, versatility, and sustainability. However, the production of ACs requires high energy input, which makes the adsorption process costly. To reduce costs and minimize waste, regenerating spent ACs is generally more practical than disposal. Among various regeneration methods, thermal regeneration, typically carried out at high temperatures, is most common in wastewater treatment. Nonetheless, thermal regeneration is not widely implemented due to high energy demands and reduced adsorption efficiency after regeneration (Sher et al. 2021). These limitations, as well as long‐term stability concerns, highlight the need for cost‐effective regeneration strategies and long‐term stability optimization to enable practical, large‐scale applications of AC‐based ion‐adsorption–photocatalytic systems in water treatment.

The effective use of clay‐like materials, such as HA and zeolite, in TiO_2_‐based composites for the removal of PHAC pollutants from water has also been investigated by several authors. In one study, TiO_2_‐HA nanocomposites achieved complete decomposition of CIP and OFL under UV light (Table 1). A later study confirmed 100% CIP elimination and ~99% TOC removal using a similar TiO_2_‐HA system (Table 1). These results could be ascribed to the complementary functions of the composite's phases: TiO_2_‐rich phases promoted efficient photodegradation, while HA‐rich phases improved adsorption (Peiris et al. 2021; Abdullah et al. 2023), probably through ion‐adsorption pathways encompassing exposed Ca^2+^ sites, surface –OH, and phosphate groups (PO_4_^3−^/HPO_4_^2−^). These interactions can facilitate surface complexation and electrostatic attractions with cationic and/or zwitterionic antibiotics (CIP, OFL), accelerating their ion adsorption.

Further development in adsorption–photocatalysis is evident in the utilization of carbon‐doped TiO_2_ coated on zeolites (C‐TiO_2_/zeolite) for the treatment of wastewater contaminated with PHACs and PST residues (Table 1). Using mordenite zeolites (SiO_2_:Al_2_O_3_; ratio of 18:240) under simulated solar light, 13 out of 18 target pollutants were completely adsorbed, possibly due to the zeolite's hydrophobic nature, slight negative charge, and large surface area which promoted ion adsorption of charged pollutants. This selective adsorption concentrated pollutants near TiO_2_ active sites, accelerating ion‐adsorption photocatalysis and enabling high degradation efficiencies (> 95%) at low catalyst loadings (0.7 g L^−1^) (Table 1). The enhanced photocatalytic efficiency can be further explained by the reduced TiO_2_ aggregation and light scattering, prolonged charge carrier lifetimes and improved e^−^/h^+^ separation due to zeolite surface acidity (An et al. 2016).

Despite these benefits, zeolite‐supported photocatalysts display significant limitations, such as pore obstruction by NOM, decreased ion‐exchange capacity at high ionic strengths, and a progressive decline in adsorption efficiency due to active site saturation. Furthermore, complete mineralization may be hindered by strong adsorption if desorption and regeneration are inadequate (An et al. 2016). Therefore, future studies should focus on developing regeneration techniques that preserve ion‐adsorption capacity without causing structural damage, incorporating hierarchical pores to reduce fouling, and modifying zeolite surface chemistry to balance adsorption and photocatalytic capacity. These developments are crucial for transforming zeolite‐based ion‐adsorbent photocatalysts into feasible and scalable solar‐powered water treatment systems.

**FIGURE 4:**
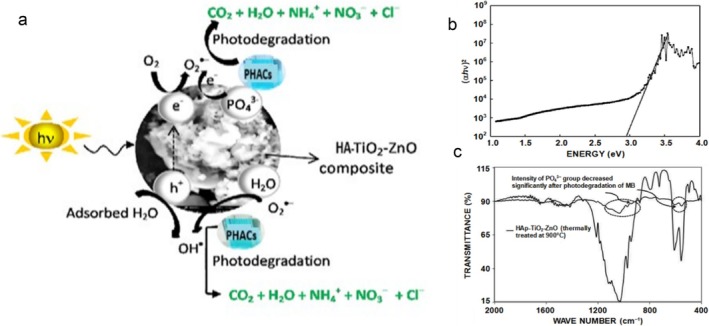
(a) Proposed PHACs degradation using HAp‐TiO_2_‐ZnO (adopted and modified from Yeasmin et al. 2018) with permission of Taylor & Francis Ltd, http://www.tandfonline.com. (b) Normalized photon energy versus (αhv)^2^. (c) FTIR spectra (before and after) of HAp‐TiO_2_‐ZnO. “Copyright permission obtained from Taylor & Francis”.

Based on previous research (Yeasmin et al. 2018), the proposed mechanism of TiO_2_‐HA composites is illustrated in Figure 4 using TiO_2_ supported on Ca‐hydroxyapatite (Hap) (TiO_2_‐ HAp‐TiO_2_‐ZnO).

The plausible Hap‐TiO_2_‐ZnO degradation mechanism is illustrated in Figure 4a. Upon UV light exposure, oxygen vacancies form at PO_4_^3−^ and HPO_4_^2−^ groups of HAp, facilitating electron capture and subsequent •O_2_^−^ production, which drives PHACs photodegradation. This process is made feasible by the low band gap energy of ≈2.95 eV (Figure 4b). FTIR analysis (Figure 4c) validates the involvement of PO_4_^3−^ and HPO_4_^2−^ groups through decreased PO_4_^3−^/HPO_4_^2−^ peak intensities, implying improved pollutant adsorption. The •O_2_^−^ reacts with surface H_2_O to generate •OH, further contributing to PHACs degradation. Noteworthy, the polar and hydrophilic nature of HAp promotes hydration‐layer formation, enhancing pollutant photodecomposition (Yeasmin et al. 2018).

## 3 Multifunctional Adsorption–Photocatalytic Nanocomposites

Table 2 shows the multifunctional adsorption–photocatalytic nanocomposites for water treatment to remove pharmaceutical residues.

**TABLE 2:** Multifunctional adsorption–photocatalytic nanocomposites for PHACs degradation in water.

Catalyst	Pollutant	Light source	Conditions	Removal (%)	References
MOFs					
Fe‐MIL‐101	TC	Visible	Conc: 50 mg/L Catalyst: 0.5 g/L Time: 3 h	97	(Wang et al. 2018)
MIP‐TC/NH_2_‐MIL‐101(Fe)	TC	Visible	Conc: 50 mg/L Catalyst: 500 mg/L H_2_O_2_: 25 μL	100	(Fu et al. 2024)
MIL‐53(Fe, Al)	TC	UV	Conc: 20 mg/L Catalyst: 143 mg/L Time: 50 min	94	(Chen et al. 2022)
CFs/TiO_2_/MIL‐101(Fe)	TC E2	Visible	TC: 20 mg/L E2: 3mg/L Catalyst: 0.2 g/L Time: 60 min	94 87	(Zhang, Xiong, et al. 2021)
Graphene‐based					
GO‐CeO_2_	DOX	LED light	Conc: 272 mg/L Catalyst: 1 mg/L Time: 7 h	100	(Abbasi et al. 2022)
KP/CN/GO/ZnFe_2_O_4_	TC	Visible	Conc: 35 mg/L Catalyst: 600 mg/L Time: 1 h	87	(Kumar et al. 2023)
3D‐2D BiOI/pCN	LVF	Visible	Conc: 20 mg/L Catalyst: 25 mg/L Time: 30 h		(Li, Yu, et al. 2021)
BiOCl/CQDs/rGO	CIP	Visible	Conc: 20 mg/L Catalyst: 25 mg/L Time: 60 min Time: 1 h	87	(Huang et al. 2020)
Biochar‐based					
Fe/Ti‐biochar	CIP NOR	Solar light	Conc: 40 mg/L Catalyst: 0.25 g/L Time: 6 h	88 88	(Cheng et al. 2023)
3c‐La_2_S_3_/SN‐biochar	TC	Solar light	Conc: 200 mg/L Catalyst: 400 mg/L Time: 2 h	98	(Peng et al. 2022)
Layered/clay‐like materials					
M‐CN/cLDH	OXC TC DXC CTC	Visible	Conc: 10 mg/L Catalyst: 500 mg/L Time: 2 h	96 97 91 74	(Yu et al. 2021)
MCC/MT‐MB	TC	UV	Conc: 50 mg/L Catalyst: 100 mg/L Time: 2 h	97 (water) > 90 (dairy manure)	(Amaly et al. 2022)
Ag_3_PO_4_‐HNTs	DCF NPX IBU FBP CBZ	Visible	Conc: 5 mg/L Catalyst: 1 mg/L Time: 2 h	n.d	(Nyankson and Kumar 2019)
Metal sulfide/oxide					
S‐scheme BiOBr/Zn_2_In_2_S_5_	TC	Visible	Conc: 200 mg/L Catalyst: 400 mg/L Time: 40 min	98	(Du et al. 2022)
SnO_2_‐ZnS	MTP CBZ ACM TCN	UV	Conc: 10mg/L SnO_2_:ZnS 2:1 Time: 140 min	67 40 70 40	(Hojamberdiev et al. 2020)
Polymer/membrane‐based					
MIP‐Ag/Ag_3_VO_4_/CN	TC	Visible	Conc: 20 mg/L Catalyst: 500 mg/L Time: 2 h	90	(Sun et al. 2019)
MIP‐BiOCl/Bi_3_NbO_7_	CEF	UV/visible	Conc: 0.8 g/L Catalyst: 1.6 g/L Time: 100 min	92	(Zhang et al. 2023)
Chitosan–glyoxal/PVP/MoS_2_	DCF	UV	Conc: 0.1 g/L Catalyst: 0.1 g/L Time: 50 min	95	(Li et al. 2019)
Ag‐doped ZnO@Fe_3_O_4_/MWCNTs (supported on PAA‐PA)	AMO	Solar light	Conc: 20 mg/L Catalyst: 1.7 g/L Time: 2 h	59	(Irani and Amoli‐diva 2020)

Abbreviations: CN: g‐C_3_N_4_; CQDs: carbon quantum dots; GO: graphene oxide; HNTs: halloysite nanotubes; MCC: microcrystalline cellulose; MIL: Materials of Institute Lavoisier; MT‐MB: montmorillonite‐hosted methylene blue; MWCNTs: multiwalled carbon nanotubes; M‐CN/cLDH: modified g‐C_3_N_4_/MgZnAl‐calcined layered double hydroxide; n.d: no data; PAA‐PA: polyacrylic acid‐modified polyamide; pCN: porous g‐C_3_N_4_/graphene; PS: sodium persulfate; PVP: polyvinylpyrrolidone; rGO: reduced graphene oxide.

Iron‐based metal–organic frameworks (Fe‐MOFs) have attracted growing attention in recent years for their application in adsorption and photocatalysis. These materials possess unique three‐dimensional architectures and beneficial properties such as high surface area, tunable pore structures, and ease of functionalization (Wang et al. 2018). The MIL series, particularly Fe‐based MILs, stands out due to the low cost and natural abundance of iron (Fe_2_O_3_), combined with strong chemical and water stability. Moreover, the presence of iron‐oxo (Fe–O) clusters imparts visible‐light responsiveness, enhancing their potential in photocatalytic water treatment (Wang et al. 2018; Fu et al. 2024; Zhang, Xiong, et al. 2021).

Among the Fe‐MOFs investigated, Fe‐MIL‐101 has demonstrated remarkable photocatalytic efficiency, achieving 97% TC removal under visible light (Table 2). In addition to strong light absorption, large surface area, and high pore volume, this removal can be ascribed to ion‐adsorptive interactions between charged TC molecules and coordinated unsaturated Fe sites, which concentrate contaminants near active sites and facilitate ion‐adsorbent photocatalytic decomposition (Wang et al. 2018). Complete TC elimination was achieved when MIL‐101(Fe) was integrated with MIP (MIP‐TC/NH_2_‐MIL‐101(Fe)) in a similar study (Table 2), further demonstrating the strong synergy between selective ion adsorption by the imprinting layer and the Fenton‐like activity of the MOF carrier (NH_2_‐MIL‐101(Fe)) (Fu et al. 2024). Bimetallic frameworks such as MIL‐53(Fe, Al) further improved TC degradation, achieving 94% removal under UV light (Table 2). These results indicate that incorporating a secondary metal (Al) can enhance crystallinity, facilitate charge separation, and extend light absorption, thereby enabling effective ion‐adsorption–photocatalytic activity (Chen et al. 2022). Additional progress has been realized through MOF‐semiconductor heterojunctions. For example, CFs/TiO_2_/MIL‐101(Fe) eliminated 94% TC and 87% E2 (Table 2). The closeness of adsorbed contaminants to photoactive Fe and TiO_2_ sites promoted direct interfacial photo‐oxidation, emphasizing the role of Fe‐MOF‐based composites as efficient ion‐adsorbent photocatalysts for the selective removal of PHACs from water.

**FIGURE 5:**
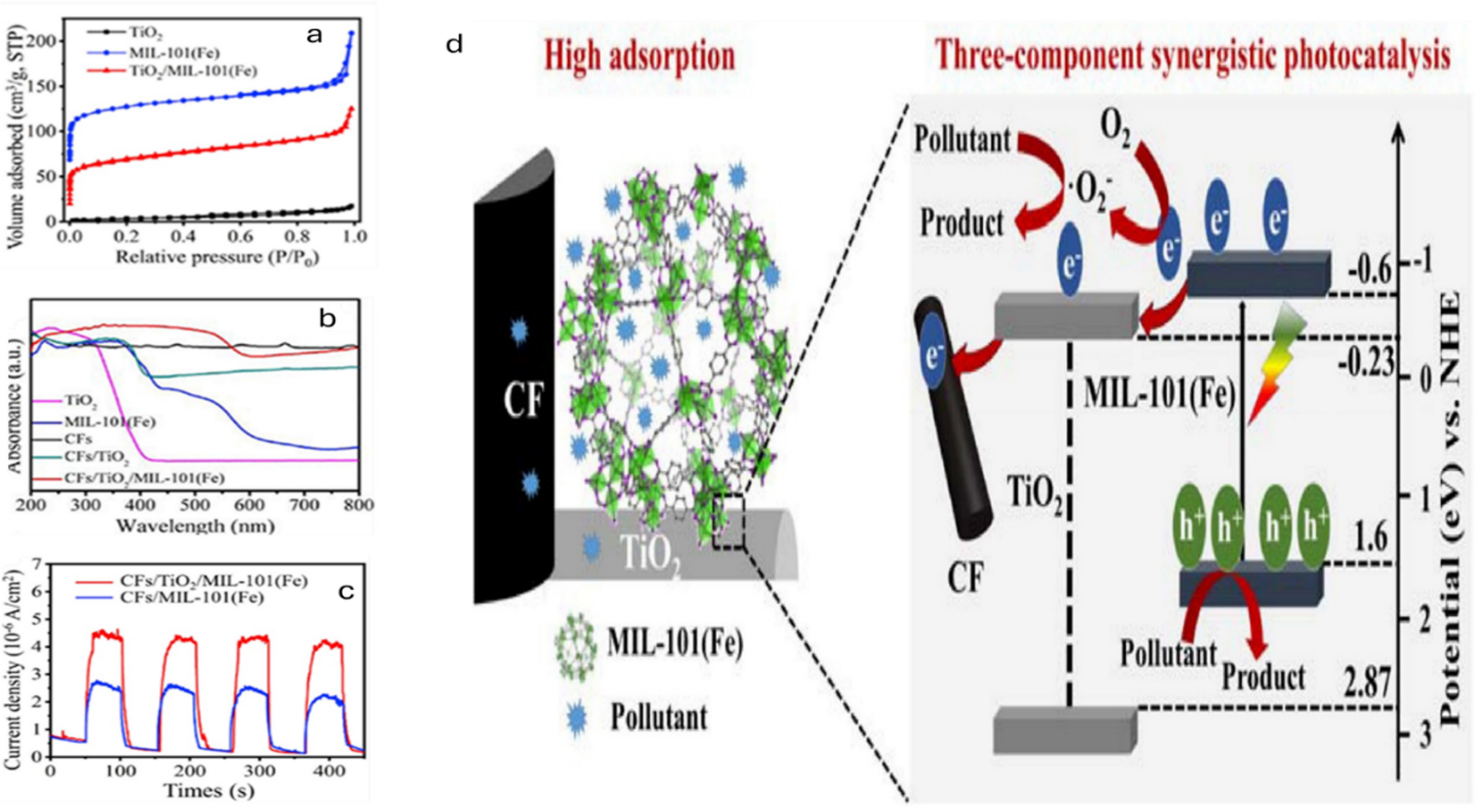
(a) N_2_ adsorption–desorption isotherms of TiO_2_, MIL‐101(Fe), and TiO_2_/MIL‐101(Fe); (b) UV–vis–NIR spectra; and (c) photocurrent responses of as‐prepared samples. (d) Proposed degradation pathway of PHACs using CFs/TiO_2_/MIL‐101(Fe) (Zhang, Xiong, et al. 2021). “Copyright permission obtained from ELSEVIER”.

However, despite the highlighted improvements, the practical application of Fe‐MOFs remains a challenge. Some Fe‐MOFs suffer from limitations such as low porosity, narrow light absorption ranges, inefficient charge separation, and instability under extreme pH conditions (Wang et al. 2018; Zhang, Xiong, et al. 2021). Additionally, complex synthesis pathways, Fe leaching, and reduced selectivity in the presence of NOM may further hinder scalability. Addressing these challenges will require material advancements that improve structural stability, photocatalytic efficiency, and ensure economic and environmental sustainability. Progressive research into hybrid ion‐adsorptive photocatalytic systems and metal doping strategies presents potential for overcoming these setbacks and advancing Fe‐MOFs toward practical water remediation applications. The mechanism of PHACs removal using Fe‐MOFs based adsorption‐photocatalysts is illustrated using the CFs/TiO_2_/MIL‐101(Fe) composite (Figure 5).

The MIL‐101(Fe) and TiO_2_/MIL‐101(Fe) exhibit high surface areas and adsorption capacities, with MIL‐101(Fe) displaying a characteristic micro‐ and mesoporous multichannel structure (Figure 5a). This framework, together with nanodot decoration, enhances the ion adsorption of charged or polar PHACs by providing abundant active sites and open transport pathways. In parallel, the three‐component TiO_2_/MIL‐101(Fe) framework delivers synergistic photocatalytic performance, as evidenced by broad photon absorption (Figure 5b) and an enhanced photocurrent response (Figure 5c), which promote efficient charge separation and transport (Zhang, Xiong, et al. 2021).

The proposed degradation mechanism (Figure 5d) illustrates that the MIL‐101(Fe) releases photogenerated charge carriers when exposed to visible light. After transferring to TiO_2_, the CB electrons of MIL‐101(Fe) migrate to the conductive CFs, where they reduce O_2_. TC and E2 are simultaneously degraded by h^+^ in MIL‐101(Fe). This speedy degradation boosts PHACs adsorption by the CFs, facilitating simultaneous ion adsorption and photocatalytic removal (Zhang, Xiong, et al. 2021).

On the other hand, graphene‐based nanocomposites, particularly graphene oxide (GO) and graphitic carbon nitride (CN), have emerged as highly effective materials in water and wastewater remediation due to their large surface area, adsorption capacity, and electrical properties. These features facilitate strong ion‐adsorptive interactions with charged pollutants, concentrating them at reactive centers and promoting photocatalytic degradation (Ajala et al. 2022). The ability of GO and CN to form heterojunctions further enhances visible‐light response and charge separation. For example, the GO‐CeO_2_ nanocomposite achieved complete removal of DOX under LED light (Table 2). This performance could have been favored by the reduced bandgap, improved charge separation at the GO‐CeO_2_ interface, and adsorption–photocatalytic degradation (Yu et al. 2021). Similarly, the PKg‐C_3_N_4_/GO/ZnFe_2_O_4_ composite showed 87% TC decomposition and 71% mineralization (Table 2), probably due to increased light response from doping, improved charge separation, and ion‐adsorptive concentration of TC on GO surfaces.

The combination of 3D, 2D, and porous structures in composites such as 3D‐2D BiOI/pCN hydrogel can increase photocatalytic efficiency. Using the BiOI/pCN hydrogel as a photocatalyst, approximately threefold higher LVF removal was obtained than for pristine BiOI, demonstrating that 3D–2D–3D heterojunction engineering promotes mass transfer, charge mobility, and ion‐adsorption photocatalysis (Cheng et al. 2023). Another notable system, BiOCl/CQDs/rGO recorded 87% CIP elimination (Table 2), significantly outperforming single BiOCl. In this system, the co‐incorporation of rGO and CQDs increased adsorption capacity and promoted superior charge generation and separation, rather than simply improving light absorption (Peng et al. 2022). These results emphasize the significance of ion‐adsorption‐driven interfacial charge transfer and utilization in optimizing photocatalytic efficiency, supporting the importance of graphene‐based ion‐adsorbent photocatalysts for selective PHACs removal.

**FIGURE 6:**
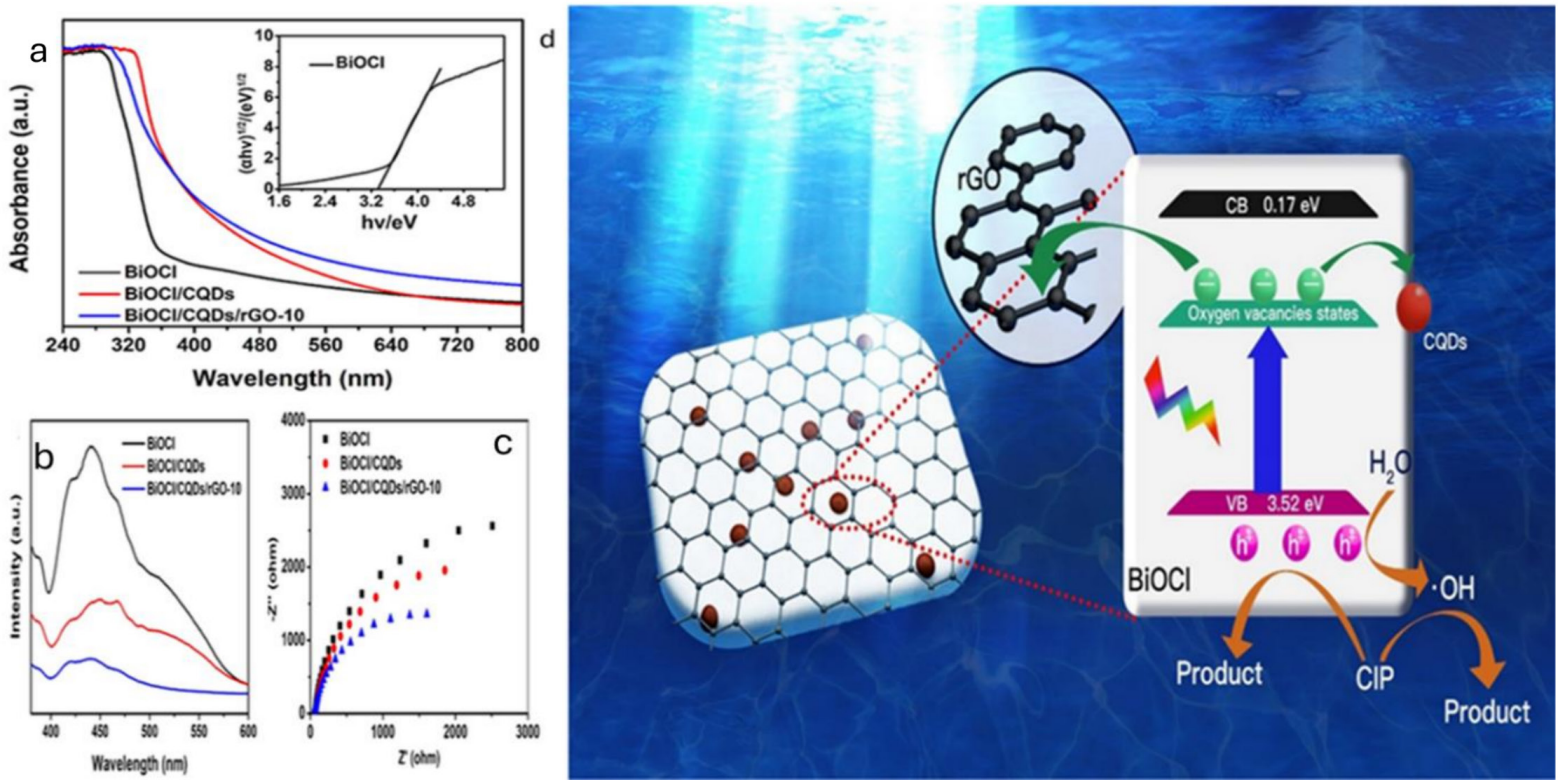
(a) UV–vis DRS, (b) PL spectra, and (c) Nyquist plots of EIS of BiOCl, BiOCl/CQDs, and BiOCl/CQDs/rGO‐10. (d) Degradation mechanism for CIP removal using BiOCl/CQDs/rGO photocatalyst (Huang et al. 2020). “Copyright permission obtained from ELSEVIER”.

Despite these advantages, graphene‐based ion‐adsorbent photocatalysts show significant limitations, including agglomeration, limited light penetration at high dosages, and suppressed selectivity in complex water matrices due to competitive adsorption from NOM and inorganic ions (Ajala et al. 2022). Similarly to carbon‐doped TiO_2_ composites, excessive adsorption on graphene surfaces can prevent complete mineralization if photocatalytic and desorption cycles are not sufficiently balanced. Future studies should therefore prioritize modulating graphene dispersion, optimizing composite ratios to prevent light shielding, and incorporating hierarchical porosity or selective functionalization to enhance regeneration and fouling resistance. Mitigating these limitations is important to fully utilize graphene‐based ion‐adsorbent photocatalytic systems for scalable and durable PHACs elimination. A typical degradation mechanism for PHACs removal using graphene‐based photocatalysts is depicted in Figure 6.

While BiOCl/CQDs and BiOCl/CQDs/rGO‐10 show broad UV/visible light absorption, pure BiOCl absorbance prevailed in the UV region (Figure 6a), suggesting that rGO and CQDs improved light‐harvesting capacity in the composites. The positive roles of CQDs and rGO in improving photocatalytic activity were further affirmed by the corresponding low PL intensities (Figure 6b) and EIS analysis (Figure 6c). These results demonstrate possible suppression of charge recombination by CQDs and rGO, which is pivotal for effective photocatalytic performance.

The CIP degradation pathway using BiOCl/CQDs/rGO illustrates that, upon visible light exposure, electrons in the VB of BiOCl are transferred to oxygen vacancy states below the CB, while h^+^ remains in the VB (Figure 6d). Because E_VB_ > E_(H2O/•OH)_ (2.27 eV), h^+^ can oxidize H_2_O to produce •OH, which drives the breakdown of CIP. Owing to their superior conductivity, electrons in the vacancy states quickly migrate to CQDs and rGO, facilitating charge separation and preventing recombination (Huang et al. 2020).

In recent years, biochar, a carbon‐rich material derived from organic waste, has attracted significant attention as a sustainable and low‐cost adsorbent for environmental applications. Its functional groups, chemical stability, and large surface area make it appealing for water remediation via ion‐adsorbent photocatalysis (Siipola et al. 2020). However, the difficult post‐treatment recovery of biochar presents the risk of secondary pollution, necessitating material modification. Recent progress involves doping biochar with transition metals (e.g., Ni, Fe, and Cu) or their oxides to impart photocatalytic and magnetic properties, enabling efficient photocatalytic degradation and easy recovery via magnetic separation. In a recent study, the Fe/Ti‐biochar nanocomposite achieved 88% elimination of CIP and NOR under solar irradiation (Table 2). This performance was probably supported by the ion‐adsorptive interactions that concentrated charged antibiotics close to Ti‐based sites, including electrostatic attraction, π–π interactions, and surface complexation (Cheng et al. 2023). The h^+^ and •OH subsequently drove the degradation process, demonstrating efficient ion‐adsorption photocatalytic elimination.

**FIGURE 7:**
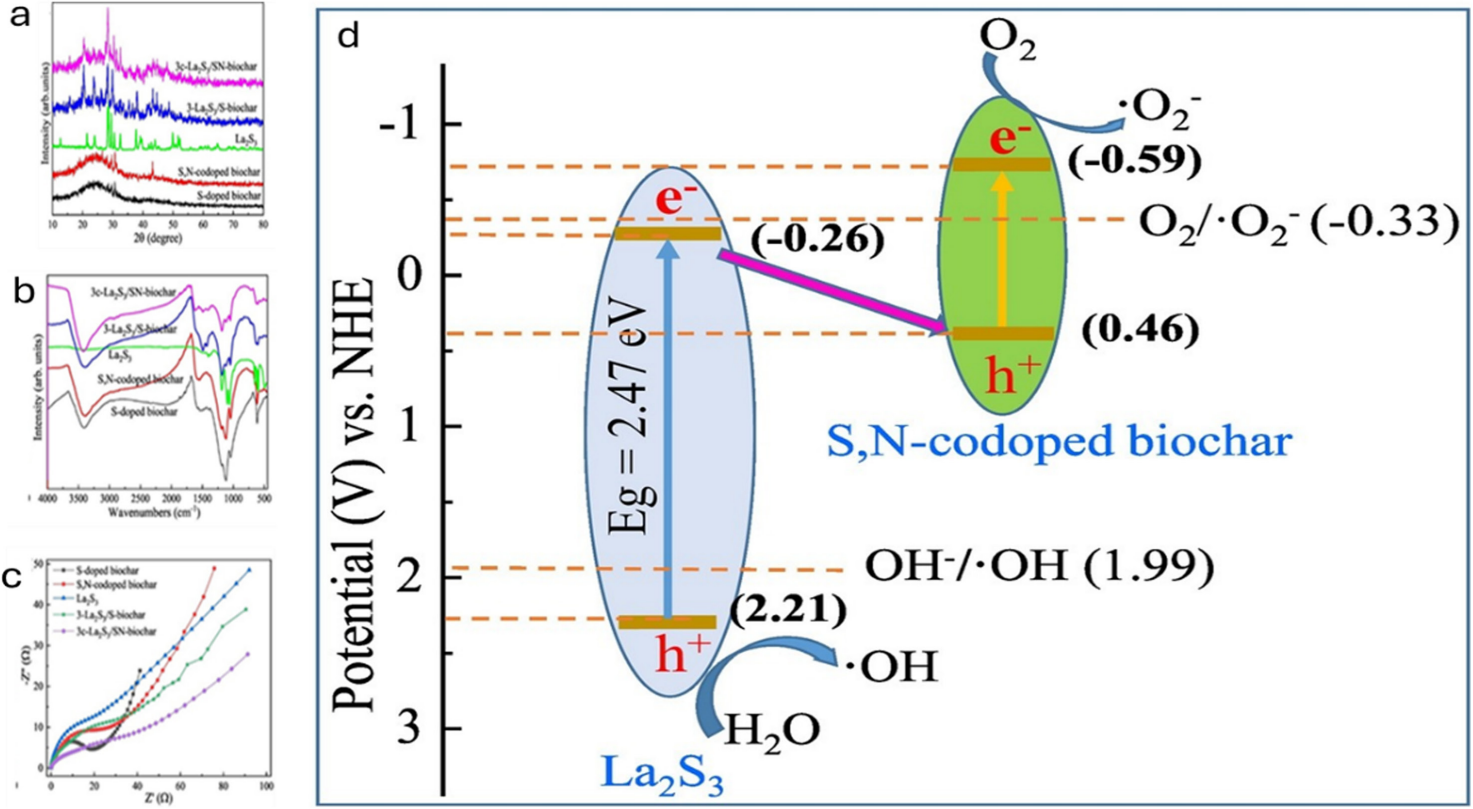
(a) XRD, (b) FTIR, and (c) photocurrent response of La2S3 and biochar‐based composites. (d) Proposed adsorption–photocatalytic degradation pathway of 3c‐La_2_S_3_/SN‐biochar (Peng et al. 2022). “Copyright permission obtained from ELSEVIER”.

In a related study, sulfur (S) and nitrogen (N)‐doped biochar modified with La_2_S_3_ (3c‐La_2_S_3_/SN‐biochar) displayed 98% TC removal (Table 2). The superior performance was attributed to favorable surface charge, multiple functional groups, and sulfur defects that boosted ion adsorption, light absorption, and charge separation (Peng et al. 2022). These findings suggest that biochar modification through metal doping and photocatalyst integration can significantly enhance biochar‐based ion‐adsorbent photocatalytic degradation. However, challenges such as photocatalyst leaching, structural uniformity, and poor performance under variable environmental conditions prevail. Therefore, further studies focusing on controlled doping strategies and robust regeneration techniques are necessary to improve durability and sustainability while minimizing environmental risks. The adsorption–photocatalytic elimination of PHACs using biochar‐based composites is illustrated in Figure 7.

Compared with the other samples, the peak intensities of 3‐La_2_S_3_/SN‐biochar assigned to the crystal planes of α‐La_2_S_3_, La_2_O_2_S, and LaS_2_ became stronger (Figure 7a), which could account for the improved adsorption and photocatalytic performance of 3‐La_2_S_3_/SN‐biochar. Figure 7b shows characteristic bands at 1300–900 cm^−1^, which represent the C–N, C–O, and C–S bonds, while the 600–400 cm^−1^ bands correspond to La–O and La–S stretching vibrations. These bonds could facilitate effective adsorption and improve visible‐light response by acting as electron traps and adsorption sites. Pollutant adsorption was enhanced by the high surface area (954.73 m^2^ g^−1^) and pore volume (0.266 cm^3^ g^−1^), while the pH‐dependent zeta potential promoted electrostatic attraction of cationic and amphoteric PHACs (Peng et al. 2022). In addition, 3c‐La_2_S_3_/SN‐biochar exhibited a low band gap (0.90 eV), attributed to interfacial interactions between La_2_S_3_ and S,N‐codoped biochar that improved visible‐light absorption (Peng et al. 2022). Efficient charge separation and transfer, demonstrated by the high photocurrent density (Figure 7c), accounted for the improved photocatalytic activity.

According to the proposed degradation pathway of 3c‐La_2_S_3_/SN‐biochar, La_2_S_3_, and S,N‐codoped biochar exhibit E_CB_ values of −0.26 and −0.59 eV and E_VB_ values of 2.21 and 0.38 eV, respectively (Figure 7d). Although the E_CB_ and E_VB_ values energetically support the formation of a Z‐scheme framework, charge separation between La_2_S_3_ and S,N‐codoped biochar is facilitated by interfacial electron transfer rather than direct band fusion or orbital hybridization. When exposed to light, e^−^_CB_ of La_2_S_3_ preferentially recombines with h^+^_VB_ of the S,N‐codoped biochar at the heterointerface, keeping the strongly oxidizing h^+^ in the VB of La_2_S_3_ and the strongly reducing e^−^ in the CB of the biochar. This indirect recombination pathway facilitates effective charge separation and mobility, mimicking the redox potential of a Z‐scheme system. Subsequently, dissolved O_2_ absorbs electrons to produce •O_2_^−^ due to the more negative CB of S,N‐codoped biochar (−0.59 eV) compared with −0.33 eV. Similarly, OH^−^ can combine with h^+^ to generate •OH because the VB of La_2_S_3_ (2.21 eV) is more positive than the •OH formation potential (1.99 eV). These reactive species promote the decomposition of adsorbed TC molecules into harmless TPs and smaller molecules over the 3c‐La_2_S_3_/SN‐biochar photocatalyst (Peng et al. 2022).

Recent advancements in photocatalytic water treatment have also focused on integrating LDHs, clay minerals, and cellulose‐based nanomaterials into photocatalytic processes to improve pollutant decomposition through synergistic ion adsorption and photocatalysis. In one study, the photocatalytic and adsorption properties of Ag_3_PO_4_ and HNTs were evaluated in a hybrid system (Ag_3_PO_4_‐HNTs) for the decomposition of various PHACs (Table 2). The composite functioned through an ion‐adsorption–photocatalysis mechanism, with Ag_3_PO_4_ acting as the photoactive component due to its strong visible‐light response. The HNTs provided selective adsorption and pre‐concentration of ionic PHACs within their tubular lumens, controlled by variable surface charge distributions (Nyankson and Kumar 2019). By increasing the availability of PHACs at reactive sites, the adsorption‐driven enrichment at the Ag_3_PO_4_‐HNTs interface facilitated subsequent photocatalytic oxidation. The Ag_3_PO_4_‐HNTs system therefore displays a dual‐functionality approach for PHACs degradation, although actual removal efficiencies were not reported.

Greater than 90% TC removals were achieved in water and dairy manure samples using an MCC aerogel composite doped with MT‐hosted MB (MCC/MT‐MB) (Table 2). The strong performance could be attributed to a synergistic ion‐adsorption photocatalytic pathway, in which the MT and MCC provided a highly porous medium and plentiful negatively charged centers for the electrostatic adsorption and pre‐concentration of cationic TC residues (Amaly et al. 2022). Simultaneously, the photosensitizing action of MB generated singlet oxygen (^1^O_2_) under light irradiation, facilitating rapid photocatalytic degradation of the adsorbed TC molecules (Amaly et al. 2022). This integrated adsorption–photocatalytic system emphasizes an innovative approach for embedding cationic dyes within clay‐aerogel frameworks to improve photocatalytic efficiency while maintaining high adsorption capacity.

In a similar study, M‐CN/cLDH was employed to degrade selected antibiotics in synthetic seawater, achieving up to 97% removal (Table 2). Through synergistic ion‐adsorption–photocatalysis, the positively charged sites and open porous framework of the calcined LDH enabled the pre‐concentration of polar antibiotic residues via electrostatic adsorption. The adsorption process improved interfacial contact with the photoactive component (CN), while the strong heterojunctions between cLDH and CN facilitated reactive species production and effective charge separation, thereby increasing photocatalytic activity (Yu et al. 2021).

**FIGURE 8:**
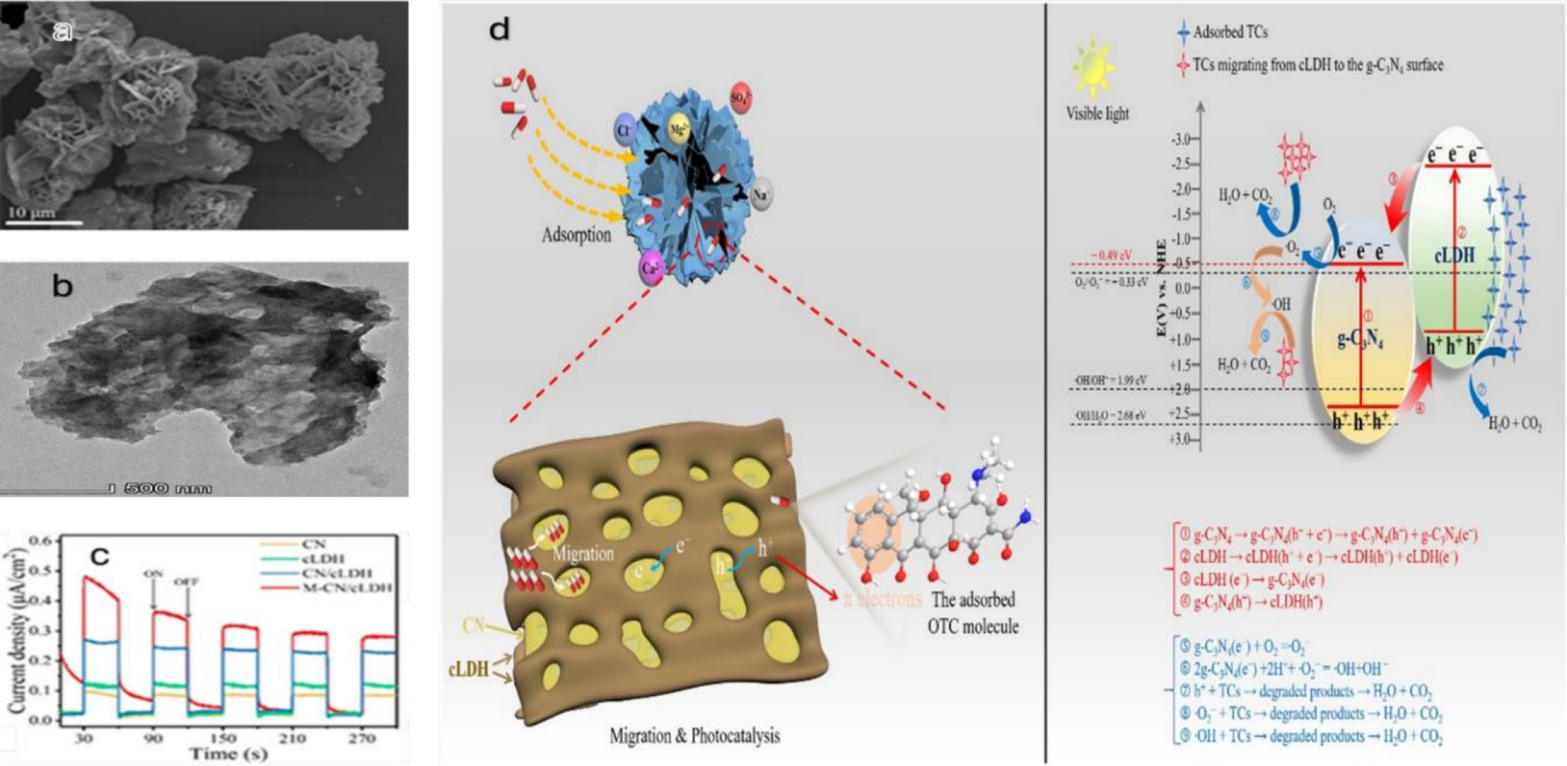
(a) SEM, (b) TEM images of M‐CN/cLDH, and (c) photocurrent responses. (d) Synergistic adsorption–photocatalytic degradation of PHACs using M‐CN/cLDH (Yu et al. 2021). “Copyright permission obtained from ELSEVIER”.

Although layered/clay‐like material‐based ion‐adsorptive photocatalysts show great promise for PHACs degradation, their practical application will depend on addressing major challenges related to scalability, selectivity in complex matrices, regeneration capacity, and composite durability. Therefore, future research should focus more on matrix interference effects, long‐term performance, reusability testing, and integration with complementary pretreatment processes. The synergistic adsorption–photocatalytic degradation of PHACs using layered/clay‐like materials is demonstrated in Figure 8 using the M‐CN/cLDH system.

The superior adsorption ability of the M‐CN/cLDH could be due to the layer‐by‐layer structures and 3D flower‐like open architectures (Figure 8a) that allowed full exposure of the 2D surface of lamellar M‐CN/cLDH. The TEM image (Figure 8b) further reveals a porous structure, hinting at a high specific surface area and additional active sites on the lamellar. Importantly, it is possible that the CN was partially covered by inorganic salt ions in synthetic sea water, while the cLDH on M‐CN/cLDH facilitated the ion‐adsorption process. Photo response analysis confirms the synergistic photocatalytic oxidation. The M‐CN/cLDH displays a higher photocurrent response than CN, cLDH, and CN/cLDH (Figure 8c), confirming faster charge separation and transfer due to heterostructure formation and template modulation.

As illustrated in Figure 8d, the degradation pathway shows that the CN‐cLDH heterojunction generates e^−^/h^+^ pairs under visible‐light illumination. Subsequently, h^+^ in the VB of CN migrates to the higher VB of cLDH, while e^−^ in the CB of cLDH transfers to the lower CB of CN. This charge separation results in the accumulation of e^−^ and h^+^ in CN (−0.49 eV) and cLDH (0.86 eV), respectively. Consequently, e^−^ in CN reduces dissolved O_2_ to •O_2_^−^, while h^+^ oxidizes adsorbed OTC, resulting in its degradation. In this system, the direct generation of •OH via h^+^ oxidation is thermodynamically unfavorable because the VB of cLDH (0.86 eV) is less positive than the redox potentials of •OH/H_2_O (+2.27 eV) and •OH/OH^−^ (+1.99 eV). Therefore, •OH is probably formed through secondary reactions involving •O_2_^−^, further contributing to OTC degradation (Yu et al. 2021).

Previous studies have also evaluated metal oxide‐sulfide composites for photocatalytic water treatment, with chitosan‐based systems emerging as multifunctional photocatalytic frameworks due to their high surface area, tunable surface chemistry, and structural flexibility. A CSG/PVP/MoS_2_ nanocomposite achieved approximately 95% DCF degradation (Table 2). This removal could be ascribed to a synergistic ion‐adsorption–photocatalysis pathway, in which strong interactions between MoS_2_ nanoparticles and the conjugated structure of DCF promoted interfacial charge transfer. Additionally, under acidic conditions, the protonation of ‐OH and ‐NH_2_ functional groups enhanced electrostatic adsorption and pre‐concentration of DCF molecules at the composite surface (Li et al. 2019). The adsorption‐driven DCF accumulation at photoactive sites favored subsequent photocatalytic degradation, emphasizing the importance of integrating ion adsorption and photoreaction systems within a single catalyst. However, performance in neutral or complex wastewaters may be suppressed due to the strong pH dependence of adsorption and the limited long‐term stability of MoS_2_ under prolonged light exposure (Li et al. 2019). Therefore, enhancing composite stability and broadening the operating pH range through surface modification and heterostructure design could improve practical applicability.

A similar study utilized ZnS‐containing industrial waste to synthesize SnO_2_‐ZnS nanocomposites, achieving up to 70% PHAC removals (Table 2). The improved removal, compared with single ZnS or SnO_2_ catalysts, was ascribed to the formation of a heterojunction, which enhanced charge separation (Hojamberdiev et al. 2020). Furthermore, the preferential adsorption of hydrated PHACs on SnO_2_ suggests increased surface area and surface activity. Variations in degradation efficiency across the target PHACs were probably caused by the interplay between photocatalyst surface properties and the chemical structures of the PHACs. However, the moderate and pollutant‐specific removal efficiencies show that surface chemistry and compound structure have a significant impact on adsorption selectivity and photocatalytic activity. Effective optimization of pore structure and surface functional groups may be necessary to increase selectivity and uniformity across various PHACs. Interestingly, the SnO_2_‐ZnS system also highlighted the importance of recycling industrial waste for photocatalyst production, emphasizing the role of such materials in environmentally and cost‐effective wastewater treatment solutions.

**FIGURE 9:**
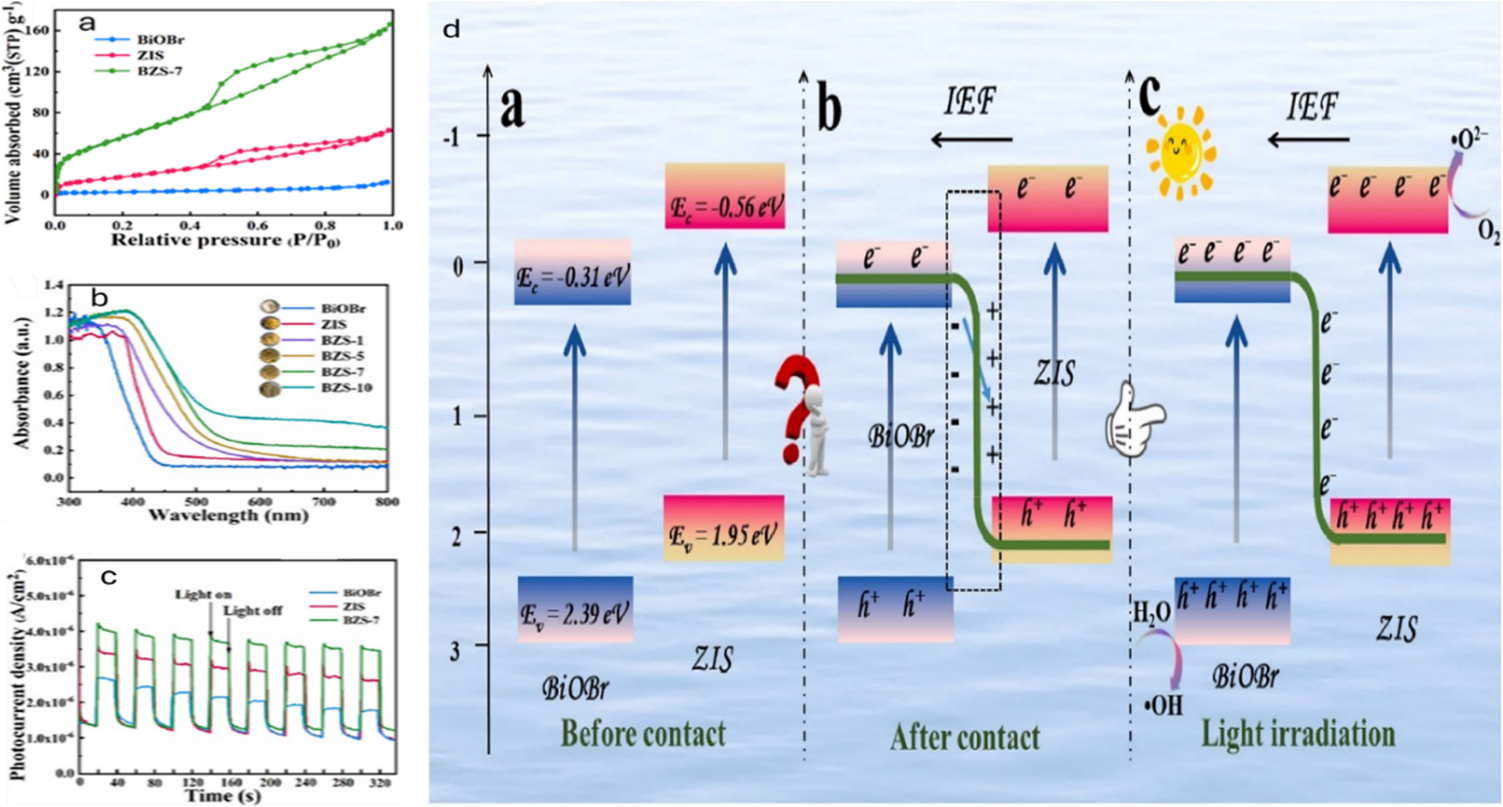
(a) Nitrogen adsorption–desorption isotherms and (b) UV–vis diffuse reflection spectra of as‐prepared samples. (c) Photocurrent curves of BiOBr, ZIS, and BZS‐7. (d) Proposed S‐scheme charge transfer mechanism over BiOBr/Zn_2_In_2_S_5_ heterojunctions (Du et al. 2022). “Copyright permission obtained from ELSEVIER”.

Metal oxide‐sulfides were further utilized in the S‐scheme, BiOBr/Zn_2_In_2_S_5_ (BZIS), achieving complete TC elimination (Table 2) under optimal conditions using the 7 wt% BiOBr/Zn_2_In_2_S_5_ (BZS‐7) composite (Du et al. 2022). The enhanced performance may be ascribed to the high adsorption capacity of the composite, possibly due to its open‐porous configuration and morphology, which provided a large surface area and abundant active sites for TC adsorption. As shown in Figure 9a, the BZS‐7 composite displayed a slightly higher surface area for harnessing more active sites for ion‐adsorption. Closely bonded heterojunctions between Zn_2_In_2_S_5_ and BiOBr layers were probably responsible for the excellent photocatalytic performance. Incorporating BiOBr improved the UV–visible light absorption of the BZS‐X composites, with BZS‐7 showing the strongest visible‐light photoactivity (Figure 9b). Furthermore, BZS‐7 exhibited a higher photocurrent density than BiOBr and ZIS (Figure 9c), suggesting that BiOBr effectively promotes charge generation and migration.

These findings may stir greater interest in the complex design of S‐scheme heterojunctions with optimized interfacial charge transfer for potential application in wastewater treatment. Nonetheless, problems with catalyst stability, scalability, and synthesis complexity still exist. Streamlined production processes and long‐term performance tests will be essential to advance the practical application of S‐scheme architectures. An S‐scheme adsorption–photocatalytic mechanism for TC degradation using the BiOBr/Zn_2_In_2_S_5_ heterojunction is proposed in Figure 9.

The proposed S‐scheme charge‐transfer mechanism begins with TC adsorption on BiOBr/Zn_2_In_2_S_5_, followed by in situ degradation by ROS under visible‐light irradiation (Figure 9d). An internal electric field (IEF) forms from ZIS to BiOBr because of the energy band alignment (Eg of BiOBr: 2.70 eV, Zn_2_In_2_S_5_: 2.51 eV). This causes band bending, which facilitates charge separation. While the ZIS remnant electrons reduce O_2_ to •O_2_^−^ and h^+^ in BiOBr oxidize H_2_O or OH^−^ to •OH, the e^−^_CB_ of BiOBr recombines with the h^+^_VB_ of ZIS. In combination with h^+^, these ROS promote effective TC decomposition. The fabrication of such S‐scheme heterojunctions overcomes challenges of single photocatalysts, including poor conductivity and rapid recombination, by boosting charge separation, while maintaining strong redox potential. This S‐scheme design draws increasing interest for improved photocatalysis because it maximizes the spatial separation of charge carriers through IEF‐induced band bending (Du et al. 2022).

The use of polymeric materials was further explored in the treatment of water contaminated with CEF using MIP‐BiOCl/Bi_3_NO (Hojamberdiev et al. 2020), achieving 92% elimination (Table 2). In this system, the MIP functioned as a selective ion‐adsorbent, and its template‐shaped pores allowed CEF molecules to be specifically recognized and pre‐concentrated at the composite surface. Through synergistic ion‐adsorption–photocatalysis, the adsorption‐driven enrichment strengthened interfacial contact with the photoactive BiOCl/Bi_3_NO, accelerating pollutant photocatalytic decomposition. Similarly, the imprinted Z‐scheme Ag_3_VO_4_/CN heterostructure removed 90% TC (Table 2). While the MIP provided selective adsorption of TC, the Z‐scheme Ag_3_VO_4_/g‐C_3_N_4_ heterostructure and the surface plasmon resonance of Ag^0^ boosted charge separation efficiency (Sun et al. 2019).

**FIGURE 10:**
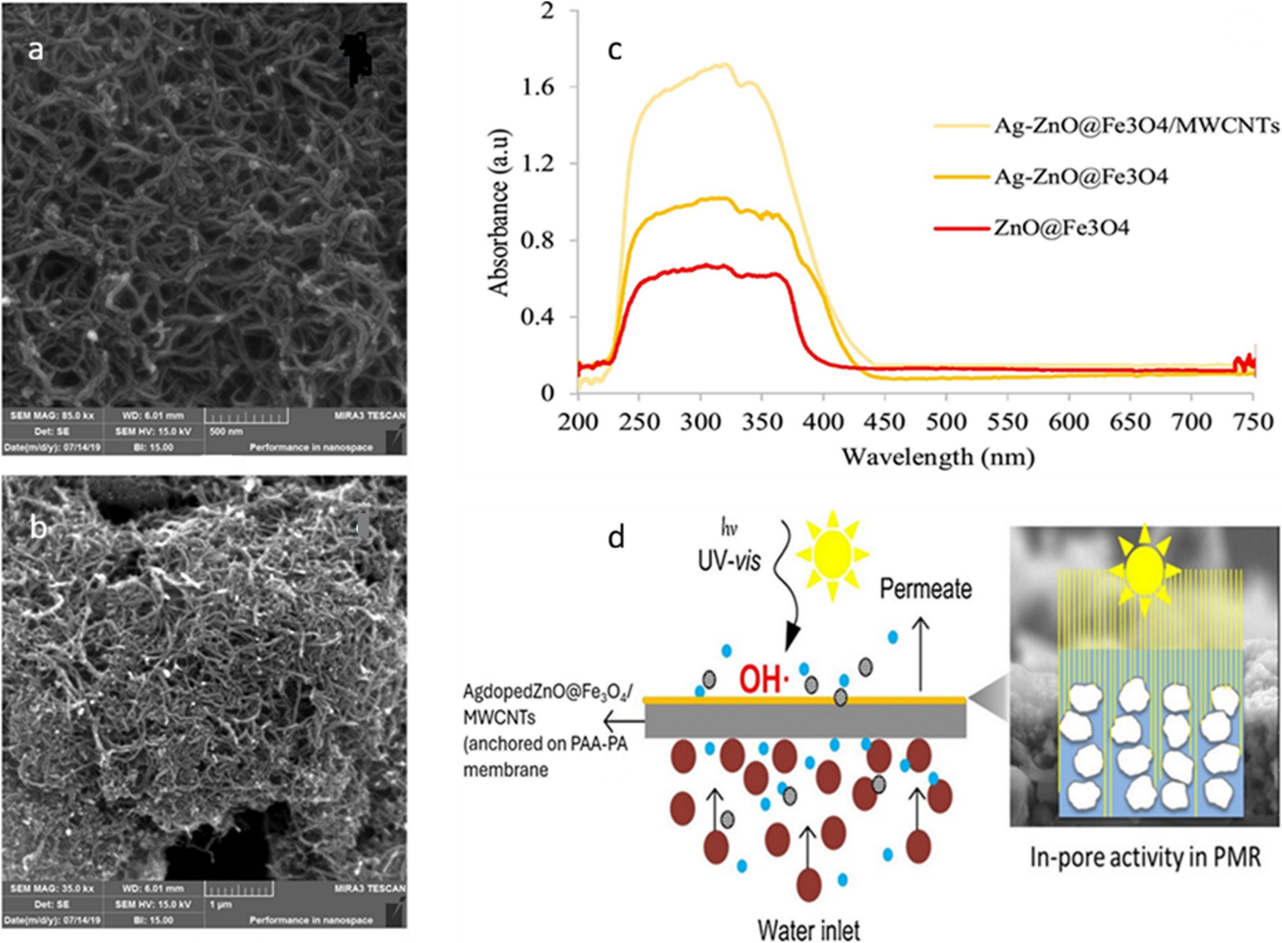
SEM images of (a) Ag‐doped ZnO@Fe_3_O_4_/MWCNTs. (b) PC‐PAA‐PA membrane. (c) UV–vis DRS plot of samples. (d) Demonstration of photocatalytic‐membrane filtration using a magneto‐plasmonic Ag‐doped ZnO@Fe_3_O_4_/MWCNTs supported on PAA‐PA (Irani and Amoli‐diva 2020; Chabalala et al. 2021). “Copyright permission obtained from ELSEVIER”.

On the other hand, membrane‐based filtration systems continue to dominate water/wastewater treatment technologies due to their operational simplicity, scalable design, and cost‐effectiveness in some cases. However, conventional polymer membranes are significantly limited by fouling, which reduces flux and raises energy demands (Chabalala et al. 2021). Surface modification has attracted much attention as a more sustainable fouling mitigation strategy by improving hydrophilicity and allowing photocatalyst integration (Pichardo‐Romero et al. 2020). Taking advantage of this, recent advancements have focused on hybrid membrane systems incorporating nanostructured adsorptive and photocatalytic components. One such system integrates magneto‐plasmonic Ag‐doped ZnO@Fe_3_O_4_ nanoparticles and MWCNTs onto a PAA‐PA membrane (Figure 10d). The multifunctional membrane system demonstrated high degradation efficiency for AMO (Table 2), as well as enhanced photocatalytic stability, antifouling properties, and sustained permeate flux (Irani and Amoli‐diva 2020). This performance can be ascribed to the incorporation of carbon nanomaterials, which enhance pollutant adsorption and pre‐concentrate AMO molecules at photoactive sites, demonstrating an effective ion‐adsorption–photocatalysis synergy within the membrane matrix. Improved surface hydration and electrostatic repulsion between negatively charged AMO and PAA functional groups further contributed to higher flux recovery and long‐term stability over repeated fouling cycles (Irani and Amoli‐diva 2020). Combining this performance with high flux, low fouling, and effective photodegradation demonstrates the potential of the photocatalytic membrane‐filtration system for cost‐effective and energy‐efficient water remediation.

Figure 10a shows uniform dispersion of Ag‐doped ZnO@Fe_3_O_4_ nanoparticles in MWCNTs and a dense, rough membrane surface (Figure 10b), favorable for improving pollutant adsorption and suppressing charge recombination. The UV–vis DRS (Figure 10c) displays improved light absorption due to band gap energy changes induced by Ag doping, while MWCNTs primarily promoted charge transfer.

The photocatalytic‐membrane filtration process removes PHACs from water by integrating filtration, adsorption, and light‐driven decomposition into a single system (Figure 10c). PHAC molecules are first attracted to and concentrated at the membrane surface by the hydrophilic PAA‐PA membrane and MWCNTs, which enhances their interaction with the photocatalyst. Under light exposure, Ag‐doped ZnO generates reactive species that degrade the adsorbed PHACs. In addition, silver improves light absorption, while Fe_3_O_4_ enhances charge transport and facilitates magnetic recovery. As pollutants are continuously degraded rather than accumulated, membrane fouling is minimized and stable water flux is maintained, rendering this system an efficient and sustainable approach for PHAC degradation.

## 4 Challenges and Perspectives

Despite the demonstrated potential of ion‐adsorption–photocatalytic systems in laboratory‐scale water treatment, several challenges hinder their practical application. One major drawback is the structural instability and metal ion leaching, particularly in ion‐adsorptive materials such as MOFs, biochar composites, and doped semiconductors. These materials often decompose under extended light exposure or fluctuating pH, resulting in diminished performance and potential secondary pollution. To address this limitation, future research should focus on developing chemically and photostable ion‐adsorbent photocatalysts, such as metal‐doped biochars or covalently linked MOF‐semiconductor hybrids, and surface‐passivated photocatalysts to improve durability under practical operating conditions.

The complex and expensive production of high‐performance ion‐adsorbent nanocomposites such as magneto‐plasmonic Ag‐doped ZnO@Fe_3_O_4_/MWCNTs membranes or MIP hybrids is another significant obstacle. Economic feasibility is diminished by multi‐step production, the use of costly precursors, and high energy inputs, particularly for large‐scale implementation. Sustainability could be greatly improved by developing low‐cost, scalable, environmentally friendly synthesis methods, including one‐pot, solvent‐free or green chemistry techniques. Simplified surface functionalization approaches using natural or waste‐derived precursors also offer a viable alternative route that maintains high adsorption–photocatalytic activity without sacrificing effectiveness.

The performance of adsorption–photocatalytic systems also falls considerably in real wastewater due to matrix interference. Matrix components such as NOM, inorganic salts, and co‐existing contaminants compete for adsorption sites, weaken electrostatic interactions, and accelerate membrane or surface fouling. These problems could be addressed through the development of selective, anti‐fouling agents, such as hydrophilic or zwitterionic surface alterations, MIPs for pollutant‐specific adsorption, and smart membrane‐based frameworks that integrate size exclusion with photocatalytic decomposition. These strategies can increase selectivity, boost active site availability, and maintain catalytic activity in complex aquatic environments.

Incomplete mineralization of PHACs and the generation of toxic TPs present additional challenges. While many photocatalytic systems achieve high degradation of parent compounds, TPs may persist longer or exhibit greater toxicity. Advanced charge‐transfer mechanisms (e.g., Z‐scheme or S‐scheme heterojunctions) that enhance oxidative degradation pathways should be integrated into future designs to ensure complete mineralization and safe effluent discharge.

Furthermore, the environmental advantages of these systems are compromised by the absence of efficient recovery and reuse strategies for photocatalysts. The difficulty of separating powdered components such as GO and TiO_2_ raises the risk of secondary nanoparticle pollution. Embedding photocatalysts into magnetic or membrane supports and fixed‐bed reactors can minimize material loss, enhance reusability, and promote safe disposal or regeneration. To guarantee environmental safety, photocatalysis must be combined with comprehensive toxicity assessments and real‐time TPs monitoring.

Finally, there is a significant gap in scaling adsorption–photocatalytic systems for continuous or commercial use. Most research remains at bench scale with limited validation in real wastewater. Integrated pilot studies and modular system design adaptable to existing treatment infrastructure are required to bridge this gap. Additionally, life‐cycle assessments and techno‐economic analyses will be crucial to evaluate operational costs, energy consumption, and environmental risks, thereby guiding the development of ion‐adsorption–photocatalytic systems as scalable, practical, and sustainable water‐treatment technologies.

## 5 Conclusion

The combination of adsorption and photocatalysis has emerged as a promising strategy for the elimination of pharmaceutical residues from wastewater, leveraging the high adsorption capacity of adsorbents with the oxidative strength of photocatalysts. Recent advancements in ion‐adsorption photocatalysis systems demonstrate the synergistic role of ion adsorption in pre‐concentrating PHACs, promoting interfacial charge transfer, and enhancing photodegradation efficiency. These systems frequently achieve near‐complete removal of a broad range of PHACs with improved selectivity and reusability. Despite these advantages, challenges such as fouling, material stability, catalyst recovery, complex synthesis, and matrix interference continue to pose barriers to real‐world application. This review outlines strategies to address these limitations, thereby providing direction for the rational design and practical application of next‐generation ion‐adsorption‐photocatalysts for water treatment.

## Author Contributions

**Pauline Ncube:** conceptualisation, writing – original draft, writing – review and editing. **Azwifunimunwe Tshikovhi:** writing – review and editing. **Sisonke Sigonya:** writing – review and editing. **Olayemi Fakayode:** writing – review and editing. **Bakang Moses Mothudi:** supervision. **Mokgaotsa Jonas Mochane:** conceptualization, supervision.

## Conflicts of Interest

The authors declare no conflicts of interest.

## Supporting information


**Data S1:** Supporting information.

## Data Availability

No new data were created or analyzed in this study. Data sharing is not applicable to this article.
