# Supplementary material for: Recent Advances in Adsorption–Photocatalytic Removal of Pharmaceuticals From Water Using Hybrid Ion‐Adsorbent Photocatalyst
Source: Water Environ Res. 2026 Mar 30;98(4):e70368. doi: 10.1002/wer.70368 (PMC13035438; doi:10.1002/wer.70368)
Supplement: Supplementary file 1 — Data S1: Supporting information. [file WER-98-e70368-s001.docx]

**Supplementary material**

**Recent Advances in Adsorption-Photocatalytic Removal of Pharmaceuticals from Water using hybrid ion-adsorbent photocatalyst**

Pauline Ncube^1^*, Azwifunimunwe Tshikovhi^1^, Sisonke Sigonya^2,3^, Olayemi Fakayode^2,3^, Bakang Moses Mothudi^2,3^, Mokgaotsa Jonas Mochane^1^

^1^Department of Chemistry, University of South Africa, 28 Pioneer Avenue, Discovery, South Africa

^2^Centre for Materials Science, University of South Africa, 28 Pioneer Avenue, Discovery, South Africa

^3^Department of Physics, University of South Africa, 28 Pioneer Avenue, Discovery, South Africa

Corresponding author: ppncube01@gmail.com

**List of Abbreviations**

ACM Acetaminophen

ACs Activated carbons

AMO Amoxicilin

CBZ Carbamazepine

CEF Ceftriaxone

CIP Ciprofloxacin

CTC Chlortetracycline

CNTs Carbon nanotubes.

CQDs Carbon quantum dots

DCF Diclofenac

DOX Doxorubicin

DXC Doxycycline

E2 17b-estradiol

EPs Emerging pollutants

FBP Flurbiprofen

GO Graphene oxide

HA Hydroxyapatite

HNTs Halloysite nanotubes

IBU Ibuprofen

LDHs Layered double hydroxides

LVF Levofloxacin

MB Methylene blue

MCC Microcrystalline cellulose

MIL Material of Institute Lavoisier

MIP Molecular imprinting

MOFs Metal-organic frameworks

MT Montmorillonite

MW Multiwalled

NPX Naproxin

NOM Natural organic matter

NOR Norfloxacin

OFL Ofloxacin

OXC Oxytetracycline

PA Polyamide

PAA Poly acrylic acid

PHACs Pharmaceutical drugs

PRL Propranolol

PST Pesticides

rGO Reduced graphene oxide.

SMX Sulfamethoxazole

TC Tetracycline

TCN Triclosan
